# Becoming ‘international’: Transgressing national identity as a ritual for class identification

**DOI:** 10.1177/14661381221082909

**Published:** 2022-08-26

**Authors:** Leonora Dugonjic-Rodwin

**Affiliations:** 8097 École normale supérieure-Paris Saclay, France

**Keywords:** embodiment, national identity, class, international schools, institutional rite, role-play games, everyday nationalism, internationalism

## Abstract

Asking how being ‘international’ relates to privilege, I analyse a role-play game, the Students’ League of Nations, where pupils and teachers from select international schools simulate the UN General Assembly in Geneva. I document distinctive practices of selection and visions of excellence as talent, using Bourdieu’s notion of ‘institutional rite’. I combine insider ethnography and quantitative analyses of the host school with a historical account of its’ elitism to bridge the gap between macro- and micro-analyses of ‘everyday nationalism’. I show how this game draws a symbolic boundary between ‘international’ and ‘local’ high schools by separating students who are considered worthy of transgressing their national identity from all others.

## Introduction

Nationalism and national identity are among those analytical categories that ethnography has redefined over the past two decades. Studies of modern nation-states, nationalism and ‘becoming national’ have largely been the object of 19th- and 20th-century historiography, and increasingly since the 1960s, the social sciences (Anderson, 1983; Gellner, 2006; Hobsbawn, 1994; Smith, 2001). Beyond significant critiques of their hegemony and top-down approach (Chatterjee, 1993; Muel-Dreyfus, 1996; Yuval-Davis, 1997), classical studies of nations and nationalism converge in showing how national identity became embedded in the habits of industrialized societies in terms of ‘re-inventing the past’. While several scholars offered sociological definitions of the nation and posed the problem of its reproduction, Michael Billig’s *Banal Nationalism* (1995: 8) provided a first systematic account **(**Özkirimli, 2000). Billig coined the expression ‘banal nationalism’ to designate ‘that which had no name’ in established nation-states and organizations, that is, the ordinary flagging of nationhood and habits of national self-identification and categorization by others. By invalidating the idea that nationalism was limited to peripheral nation-states and extremist organizations, he argued that it was being reproduced by well-established states, organizations and individuals through a continuous display of practices, beliefs and habits – without being recognized as such. Considered a seminal text of a subfield of nationalism studies, the thesis of banal nationalism has taken an ethnographic turn over the past 15 years as the focus shifted to the study of ‘everyday nationhood’, or how ordinary people participate in the production and reproduction of the nation (Fox and Miller-Idriss, 2008). Scholars distinguish between nationalism and ethnicity as categories of analysis and practice (Brubaker, 2013). Nationalism and ‘being national’ are considered neither good nor bad in themselves, but rather as a condition of our times (Eliassi, 2014); ethnography is viewed as providing access to the ‘cultural intimacy’ of individuals’ national understanding of themselves (Herzfeld, 1997).

In contributing to this paradigm shift toward a bottom-up approach of nationalism in practice, I combine insider ethnography with quantitative and historical analyses to bridge the gap between macro- and micro-approaches of ‘everyday nationalism’ (Hearn and Antonsich, 2018). I propose a case study of the SLN, a role-play game where students and teachers – from select ‘international’ high schools worldwide – simulate the UN General Assembly at the UN headquarters in Geneva. Dating from the 1920s, such role-play games, more widely known as Model United Nations have become a common form of educational practice since the 1960s with the creation of the International Simulation and Games Association in the Netherlands and academic journals such as *Simulation and Gaming* (Muldoon, 1995; Rosenthal et al., 2001; Shannon, 2020).^1^ While the state-of-the-art on this topic revolves around teaching politics and international relations, much existing literature aims to improve the outcome of role-play simulations as tools for education and decision-making. In contrast, I provide a sociological perspective and use ethnography to show the relationship between sentiments and acts (Khan, 2017).

First, based on everyday nationalism literature and my previous work on international education as a social field, I question the conventional notion that internationalism excludes or constitutes an alternative to nationalism in practice. Second, using one of Pierre Bourdieu’s less used and abused concepts, that of ‘institutional rite’, I examine how banal nationalism is embodied in an intensive setting for socialization whereby privileged youth are dubbed ‘international’, on the basis of their ability to transgress their national identity. Third, I propose a mixed-methods approach that empirically reveals the intuitive and unreflective dimensions of this role-play as socially distinctive. In summary, I consider the SLN as a case for investigating ‘everyday nationalism’ and understanding the role and symbolic power of transgressing national identity in the process of educating an ‘elite’.

## Theoretical framework

Cutting across several areas of sociology: domination, education and globalization, the literature on internationally mobile groups, whether they are conceptualized broadly as ‘elites’ or ‘migrants’ – is typically silent when it comes to understanding the paradox of internationalism, that is, the persistence of national schemes for self-identification within international spaces. Sociological studies of international phenomena have documented the predominance of national logics, hierarchies and asymmetries, thereby providing extensive evidence against the discourse on the decline of nation-states, be it in the name of cosmopolitanism, internationalization or globalization (Calhoun, 2007; De Swaan, 2001; Sassen, 2007; Wallerstein, 1997). Yet few have entirely suspended the common belief that when living abroad some national groups are ‘expats’, ‘international’ and ‘cosmopolitan’, while others are ‘immigrants’, ‘communitarian’ and ‘postcolonial’. As Loïc Wacquant once put it: ‘(…) how can one not see that those who are designated – indeed defamed – across Europe as “immigrants” are foreigners of postcolonial origins and lower class extraction, while others of upper-class standing are “expats”, whom everyone wants to attract (…)?’ (Wacquant, 2014).

Waquant points to a blind spot in the literature, especially studies focused on elites. Indeed, what makes some individuals ‘immigrants’ and others ‘expats?’ In his study of Algerian immigration to France, Abdelmalek Sayad found that the peasants who had been deracinated as emigrants tended to become sub-proletarians when they immigrated (Sayad, 2004: 91). Based on a study of international schools in the Paris region, Anne-Catherine Wagner found that where social class is relatively homogenous, national identity becomes the principle of social hierarchy (Wagner, 1998). Confirming Wagner’s findings, I observed that students’ nationhood was the first marker of self-understanding and identification by others in international school environments beyond the French case. Moreover, I have used such schools as ethnographic sites for examining, in a socio-historical perspective, how being ‘international’ designates a way to embody privilege. Based on previous work, I conceive the international identity asserted by these schools as a way of enacting a social difference by valuing the diversity of nationalities and national identities that students are ordinarily said to ‘represent’ (Dugonjic-Rodwin, 2014). I therefore analyse the SLN within a broader investigation of international education as a global field,^2^ defined as a sphere of specialized practice organized across continents on the basis of specific institutions and categories of perception (international/national) that have historically defined its boundaries in contrast with national education systems (Dugonjic-Rodwin, 2014, 2021). In a field-analytic perspective, rites are ‘institutional acts’ (Bourdieu 1982: 61)^3^ constitutive of lasting dispositions. Within this global field, I view the SLN as a ritual, that is, a set of regulated symbolic practices that initiate students to become ‘international’. This includes rules of the role-play game and rites such as the ‘debate’ at the *Palais des Nations*. Supervising teachers define the debate by prescriptions on how students should transgress their national identity (cf. Supplemental Annex, SLN rules, p.11-14.) and evaluate students accordingly.

Because social scientists’ criteria for identity and community (regional, ethnic or national) are themselves objects of representations in social practice (Bourdieu, 1980b), I consider national identity as a way to view the world according to ethnic boundaries (Brubaker et al., 2004). Such symbolic boundaries may be defined as a subjective sense of belonging ‘based on a belief in shared culture and common ancestry’ (Wimmer, 2008), with reference either to cultural practices perceived as ‘typical’ for the community, or to myths of common historical origin or phenotypical similarities.^4^ Following Wimmer, I consider ethnicity to subsume race and nationhood, which is predominant in such a school setting. Contrary to the discourse of political internationalism – which prescribes neutrality and sometimes even opposition to nationalism in the specific logic of the global field – the ethnographic approach reveals how internationalist practice cultivates the everyday belief in national identity and habits of national identification. This is hardly surprising because historically, the rise of nationalism entailed the invention of internationalism (Robertson, 1997; Ruyssen, 1961). We know that the creation of national identities by means of cultural institutions and modern education systems was an international process involving emulation among nationalists (Thiesse, 1999). The consciousness of national identity assumes an imagined ‘international community’, which is both the condition for and the product of national categories of perception (Billig, 1995: 83).

## Materials and methods

I attended the role-play game as an apprentice sociologist conducting a historical and ethnographic study and learning hands-on to ‘render the familiar strange’ (Mills, 1959) as I had been a student at the school 7 years earlier (cf. Journal in Supplemental Annex). I initially conducted research on the International School in Geneva (or ‘Ecolint’) between 2006 and 2008, spending 2 months on the premises. My immersion included reviewing archives, retrieving statistics, interviewing two dozen teachers in French and in English, and participant observations of classes as well as special events such as the graduation ceremony and the SLN. Using ‘ethnography by distancing’ (Beaud and Weber, 2010; Weber, 2017), these were recorded in a fieldwork log, some of which are rewritten and reproduced in Supplemental Annex. Experiencing a controlled return to one of the sites of my secondary schooling, I was conducting ‘insider ethnography’. Being an alumna, other than formally giving me rights, facilitated obtaining the authorization to investigate and make contacts during fieldwork. This is how I gained access to a notoriously closed social universe often described as a ‘bubble’ by locals.

Once on the spot, I noted that my internal point of view contrasted with the external viewpoint I was developing (cf. Journal in Supplemental Annex). I could compare this lived experience with my sociological observations. It also fed into my interviews with Jean-Marc and Claude, who initially conceptualized the rite in its current form and who voiced, as a result, the significant changes that the regulations did not record. My student memories served as an interviewing technique inviting interviewees to reflect on differences between then and now. The mixed-methods approach was an outcome of a reflexive ‘socio-analysis’ (Bourdieu, 2004). It was a distancing method, where the first step was to understand what separated me from the universe that I was investigating (Elias, 1978). As a product of the School, historicizing the institution and its rites enabled me to include my ‘school unconscious’ in the object of study thereby using social science as an ‘instrument of self-appropriation’ (Wacquant, 2004).

I have found it valuable to re-analyse ethnographic data collected over a decade ago, having not only gained distance from the object but also from the plethora of studies on internationalization to which I had previously contributed.^5^ I could see students ‘representing’ nations, as it is said at the School, but from a different perspective. It was like an experiment: in contrast with the everyday uses of nationhood that the School ordinary amplified by being ‘international’, here was a selected group of students gathered in an assembly hall at the *Palais*, where the rules of the ‘Assembly’ prohibited them from representing their ‘country of origin’. In hindsight, the SLN was a case for investigating ‘everyday nationalism’. I examine what the supervising teachers require of students in this ceremonial context and observe how students react to this extraordinary situation.

First, to provide a sense of the School’s broader context, I draw on excerpts from five interviews, historicizing the rite based on archival documents and statistical analyses of the students’ social characteristics. I then use journal notes, document analysis, observations and interviews with the supervising teachers and organizers of the SLN, Claude and Jean-Marc, to analyse the SLN as an ‘institutional rite’. Rather than using interviews as illustrations or empirical evidence for arguments, I use them as one source among others to construct the SLN as a socio-historical object of analysis. I report extracts anonymously to shed light on the practices observed, the social composition of the School,^6^ as well as on my memories of personal experience.^7^ Finally, I discuss how the rite inculcates a way to embody the privilege of being ‘international’.

### Social composition and role of national identity

Ecolint hosts the SLN annually. Today, the School enrols approximately 4450 students of more than 100 nationalities on its three campuses.^8^ The director of Education at the time of the survey, Marwane, summarizes the social composition of the School well:

‘When you look at the International School, in their number, there are no lower classes. There are the upper middle classes and the upper classes. (…) So what we lack in the educational apparatus of this school is the contact, the inter-social mixing. We have intercultural mixing, but we lack inter-social mixing’.

**Figure 1.**
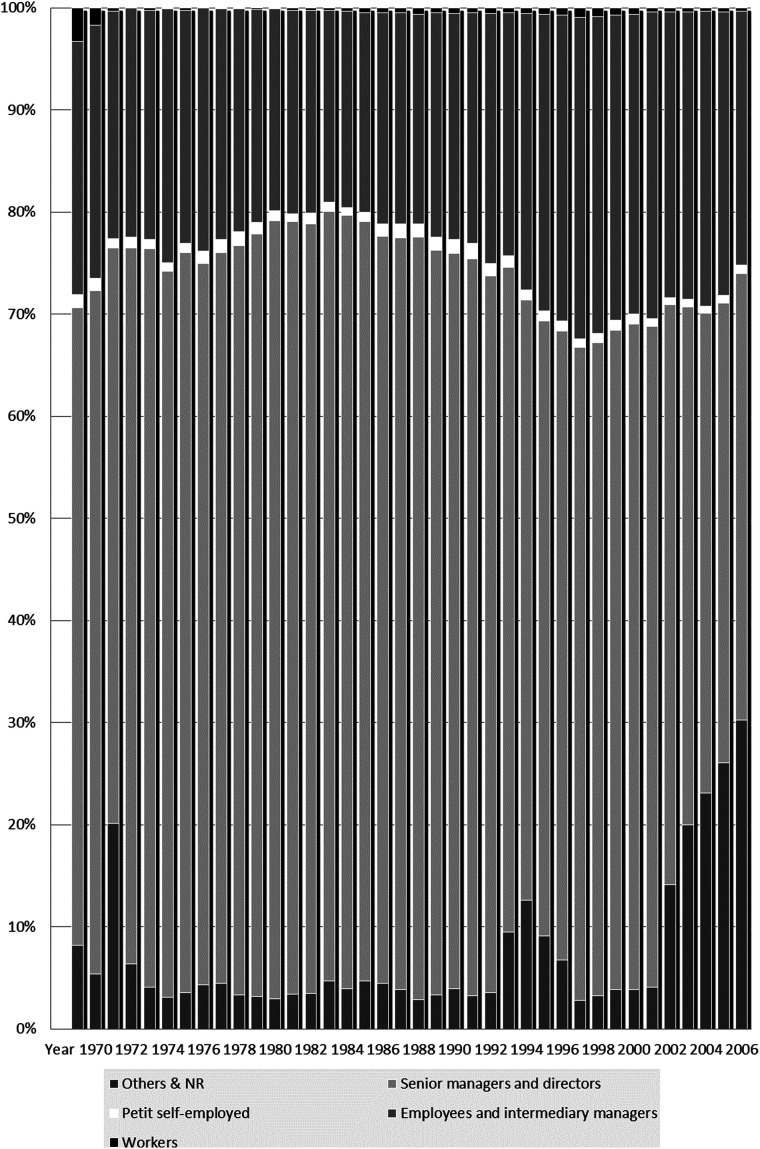
The Social Composition of Ecolint, 1969-2006. Source: SRED.

Public statistics compiled by the Educational Research Service (SRED)^9^ with an average response rate of 90%, confirm Marwane’s portrayal, providing a long-term perspective (see Figure 1 below). Between 1969 and 2006, ‘senior managers and directors’ were best represented at Ecolint. The extremely low share of ‘workers’, that is, employees in professions defined as ‘manual’, and the ‘small self-employed’ is constant over this period of close to 40 years: they each constitute on average 1% of the population.

The graph shows that what the natives call the ‘international community’ excludes these two categories primarily based on the School’s registration fees. The price of Ecolint for the year 2019–2020 varies between CHF 20,370 [$ 20,603] in nursery classes and CHF 34,950 [$ 34,961] for classes corresponding to the first and final year of high school.^10^ Yet there are three options for enrolment in the School. If the father^11^ works for a multinational company as an expatriate, his employer covers everything. If he works for an international organization at the professional level, he pays only 25% of the fees, and the rest is reimbursed under the UN Education grant.^12^ If the father belongs to the aristocracy or the local bourgeoisie, especially if he exercises one of the professions, he must fully cover the fees. There are therefore three categories of clients at Ecolint: (1) the local aristocracy and bourgeoisie, who pay 100% of the registration fees directly, (2) international civil servants, who pay 75% of the registration fees directly but are reimbursed, and (3) expatriates for whom the school is free because their companies fund the School through donations (Dugonjic-Rodwin, 2022: 239).

To recruit students, Ecolint depends on international organizations and multinational companies based in Geneva. Through the mediation of students’ fathers, these two categories of employers constitute on average three quarters of its clientele (see Table 1 above). Neither artisans nor traders, those that compose the category ‘independent’ are above all professionals like lawyers, doctors, architects, psychoanalysts, company directors and self-employed executives. Despite the difficulty to compare initial social positions among their countries of origin, the members of this ‘international of national elites’ (Dezalay, 2004) occupy a dominant position in their country of destination, in this case Switzerland. If the father’s sector of activity highlights internal differences in terms of class fractions, values and lifestyles, Ecolint is socially homogeneous: it is overall a school tailored for the dominant classes.

**Table 1.** Distribution of students promoted in 1976 and 2000 by father’s sector of activity.

Father’s sector of activity	1976 (n = 150)	2000 (n = 128)
Private sector	45 %	48 %
International civil service	29 %	19 %
National civil service	9 %	13 %
Independent	11 %	15 %
Non-response	5 %	5 %
Total	100 %	100 %

*Source*. Author’s database, Ecolint 1969 and 2000.

Breaking with national educational traditions when it was established in 1924, the School claimed an ‘international spirit’ with reference to Wilson rather than Lenin, and more broadly to the political ideology of the League of Nations (Dugonjic-Rodwin, 2022: 135‐6). Today, as in the past, references to national identity are omnipresent in school publications, official discourse and tittle-tattle. School statistics count nationalities to measure the international character of the institution and annual reports publish these numbers. Students are said to ‘represent’ more than a hundred nations. Ritually recalled in self-presentations, nationalities are even marked under their portraits in yearbooks, on the food their mothers prepare for the ‘kermesse’, and on their graduation outfit. Students grow accustomed to identifying themselves as belonging to their ‘home countries’ and to identifying their classmates according to their ethnicity based on either race or nationhood (cf. Theoretical framework).

While nationalities play a prominent role in classifying staff, students and parents at this school, they are not all equal. There is a concordance between the objective hierarchies of states and the historical division of the School into ‘sides’ according to the status of languages. Students are required to study in English and French, the most legitimate languages officially used by the surrounding international organizations. The School relegates all other languages to the extracurricular context of private lessons, provided it can find an adequate teacher. Between these two extremes, German, Spanish and Italian are taught at the School as options.^13^

The linguistic and geopolitical relations between states are ‘refracted’ in the hierarchy of languages and nationalities at the School that has directly observable effects on curricula and teaching practice (Dugonjić and Richard de Latour, 2014). Refraction is the key notion here. In a field-analytic perspective, it refers to the ability to transform external influences or constraints into internal operating norms and logic (Bourdieu, 1969). While ethnic identification may have observable effects on social relations, it does not mean that it mechanically reproduces power relations between national groups in direct daily interactions. Indeed, even before specifying students’ gender, teachers designate their national identity. Newer teachers are impressed with the number of nationalities ‘represented’ by the students in their class, identifying this diversity with abstractions such as the ‘whole world’, the ‘international community’ or a ‘bazaar of all nationalities’.^14^ Such irenic visions of the world do not prevent them from perpetuating stereotypes. Asians, Koreans, Indians and Japanese from ‘not very wealthy’ families study to ‘climb up the social ladder’. They ‘do first and then understand why’, whereas ‘people from the West’ ask too many questions: ‘They want to know first why, and then they act’.^15^ In examples teachers take, the ‘African’ student is typically the one punished by his parents for a bad grade while the ‘American’ challenges the teacher’s authority, certain of being worth a good grade.^16^ In situations of interaction, the nation that students ‘represent’ at the School can be stigmatized or emblematic depending on how the geopolitical context refracts onto the student’s social background. Such explicit references to geographic origin in countries of immigration implicitly designate social relations in the countries of emigration (Sayad, 2004). Whereas the discourse of political internationalism prescribes and declares neutrality, practices–ranging from the classification schemes based on language and nationhood to the recruitment of these professionals (David-Ismayil et al., 2015)–refract the established social relations between nation-states.

### The Students’ League as an institutional rite

Every year, about 100 high school students aged 16 to 18 travel from their classrooms to an assembly hall at the prestigious *Palais des Nations* for two intense ‘working days’, during which they simulate ‘delegates’. To prepare the role-play, they write ‘resolutions’ on current topics such as global warming, the proliferation of weapons of mass destruction or anti-terrorist measures, from the perspective of their country of choice. On this occasion, the School violates the very principles of its activity in ‘international education’, which is to inculcate in students a vision of themselves as individuals grounded in national identity and ‘be faithful to their convictions’.^17^ Conversely, the role-play values the students’ ability to (1) represent a nation other than their own and (2) hold a speech contrary to their conviction.^18^ Therefore, one may ask, why solemnly prohibit ‘delegates’ from representing their countries?

‘– That’s the most basic rule of all. – What is the significance of this rule to you? – (…) even Ecolint students are often emotionally involved with their countries of origin and if their countries were under attack they would get upset and that’s not what we want and also the educational purpose is really to do research into a country that has nothing to do with yours and to understand their perspective’.^19^

This directive is decisive in the role-play game. More than a school tradition, the SLN thus affirms itself as an ensemble of ‘institutional rites’. As such, it aims to change the image that these students have of themselves and the way others perceive them. Bourdieu warns that one should not be fooled by the rite which ‘draws the observer’s attention to the passage […], whereas the important thing is the line’ (Bourdieu, 1982: 61). If we move the gaze from the act of passage to the boundary that the ritual makes the students cross, we do not see the same thing. Focusing on the passage, the rite differentiates the high school students who have undergone it from those who have not yet. If we question the social significance of the boundary, however, the rite separates. Based on the ‘resolution’ that the students write, it places those who are eligible to participate in the SLN on one side of the boundary, distinguishing them from those who are not eligible and never will be. We may thus define the SLN as a school ritual that discriminates students based on their capacity to write a UN-style ‘resolution’.

‘There is a selection process for which they have to write responses to old resolutions from the perspective of a country that they will not necessarily represent and then on the basis of the quality of those responses, we establish a hierarchy’.^20^

According to the quality of their resolutions, there is a better chance students will get one of their three countries of preference. Students tend to seek the countries that are central to the SLN debates, Claude explains. These are the Security Council countries (China, France, Russia, United Kingdom and United States), Cuba, North Korea and Israel. This is only the first stage in the selection process, which we should consider as part of a larger historical process of segregation from all other Genevan high schools.

The name of the ritual has a conflictual history. It was born in the early 1990s from the split with an older school ‘tradition’ called ‘Students’ United Nations’ in English, which everyone calls the ‘SUN’. Robert J. Leach, a history teacher at Ecolint, designed it in the decade following the Second World War (see Photo from 1959 below). He aimed to help high school students understand how the UN worked. Today both role-play games exist in Geneva but capture a different audience.

In the words of Jean-Marc, the SUN ‘began to deteriorate, to be less well...managed’.^21^ Having been involved in supervising the SUN, Claude recalls: ‘The whole thing was a mockery. They would pretend to wear national costumes, which were just grotesque caricatures. Essentially they were making fun of the countries they were supposed to be representing. (…)^’22^

In 1993, the Director General of Ecolint decided that the students would no longer participate, at the cost of not organizing anything for a year and remaking ‘something better’. He recruited four teachers to write the rules for the current role-play, including Jean-Marc and Claude. Nevertheless, they claim to be the heirs of the SUN, as the first simulation of the UN General Assembly, to better distinguish themselves from it today.

Why leave a ritual that originated at Ecolint itself to remake it? The most significant differences between the two role-play events are their audiences, organization and locations. The SUN had gradually expanded from the 1970s to include Geneva and public *collèges*^23^ from other Swiss cantons. As for the SLN, it is almost restricted to Ecolint, which invites a small circle of elected schools to participate. While the SUN is a full-fledged association, the SLN is an extracurricular activity, like the choir or sports team – something that selected students do on the side and on a voluntary basis. While one occurs on the outskirts of Geneva’s international district – ‘the *Centre des Conférences*, which is a big scale venue, an expensive one to hire’^24^ – the other takes place in the heart of the district, in the *Palais des Nations*.

That Ecolint should withdraw from a school tradition that it created would intrigue any observer. Under these conditions, the name change is not surprising in itself. What is strange is that the League of Nations replaced the United Nations yet it is the UN General Assembly that is simulated and this in a ‘realistic’ way. Both refer to the privileged relationship of Ecolint with these international organizations, which are themselves part of a historical continuity.^25^ However, the School is more closely connected to the League, having been initiated by a handful of its officials in the 1920s with the support of educationalists such as Adolphe Ferrière who conceived the school as a ‘miniature League of Nations’^26^ (Dugonjic-Rodwin, 2014). International schools originated in the context of the ‘institutional turn in internationalism’ (Rasmussen, 2001). They were established as international to meet the special needs of professionals newly recruited to international civil service and their legal status as a distinct professional group whose allegiance to an international organization involved cultivating a degree of autonomy from their nation-state (Dugonjic-Rodwin, 2014; 2015).

Paradoxically, the organizers highlight this affiliation that preceded the 1950s, and thus the very origins of the SUN, of which they claim to be the heirs. With reference to the League of Nations, it presents itself as the most suitable to organize a simulation of the United Nations. By referring to its social origins, Ecolint therefore reaffirms its elitism on the occasion of the split. From the moment the simulation is limited to Ecolint and changes its name, it may be conceived as a ritual involving what Bourdieu called ‘institutional rites’. From a sociological perspective, its major effect, as a ritual, is to separate the students from Ecolint from all other high school students in the region. If the aim remains the same, the ritual grants exclusive privileges to the ‘young people’ whose parents can afford to educate them at Ecolint. In view of what has become of the SLN and of SUN, the School has withdrawn from the SUN to create a more elitist event. It is as if its new name could itself define the social and mental boundaries that must be protected at all cost by cutting itself off from all other schools.

Ecolint’s search for confinement within the same social universe reinforces the social significance of its withdrawal. The list of invited schools^27^ shows the desire to integrate an international dimension not only by means of location, but also in terms of vocation. Invited schools are mostly English speaking and almost all located in wealthy nations (Canada, the Netherlands, United Kingdom, Switzerland, France, Kuwait and Norway). Their international vocation is often present in the name itself.

Extended to public colleges in the 1970s, the SUN enlarged its working languages by adding German. While the SUN thus internationalizes from a linguistic point of view, it regionalizes in terms of student selection. Ecolint therefore appears as one of several Genevan high schools, which is a considerable downgrade in view of its privilege. In brandishing its international vocation as the only historically legitimate one, the School has always sought to distinguish itself from ‘national’ and ‘local’ schools (Dugonjic-Rodwin, 2014; 2021). Its withdrawal suggests a preference for mingling its students with those of other international schools rather than those of relatively less elitist public colleges.

In short, the School separated from the SUN in the 1990s and thus excluded all other high schools from a privilege that only it could obtain, that is, access to the *Palais des Nations*. In practice, Ecolint’s elitism sharply contrasts with the generality of the aim it sets for itself. In reality, the simulation evolves towards a form of internal cultural confinement rather than being open and welcoming to ‘all young people’ in the region. Yet this history remains in the archive and in the memory of those who witnessed the split, while official documents present the SLN as ‘motivated by the search for peace and social, economic and moral progress in the world’.^28^ Claude explains that the last 10 years is a very short time from the perspective of over 30 years of experience with this role-play game (the SUN and the SLN):

‘Not much has happened, it pretty much replicates itself… I mean, from a supervising teachers’ point of view it becomes… not dull, I’m not disenchanted with it, but let’s say that they all kind of merge one into one another… I am not aware of any major development. We have tinkered with the rules and regulations. Basically they work so “if ain’t broke why fix it”’.^29^

### Distinction: A role-play unlike any other

In the interview, Jean-Marc explained that they were trying to create something different from what I had experienced shortly after Ecolint had withdrawn from the SUN. He insisted on this desire to create a ‘totally different atmosphere’ that he called ‘professional’, thus giving me the ‘little secrets’ that contribute to it. First, the location: ‘it’s not the same as organizing a simulation in a school’, he explained to distinguish the SLN from the myriad of Model UNs practiced in the United States. Being at the *Palais*, surrounded by ‘real delegates’ they meet in the corridors, is supposed to encourage students to take the role-play seriously. Second, these students are not novices; some are even ‘regulars’. Indeed, some teachers bring their students two or 3 years in a row: first ‘to see how it goes’, and then ‘they become a major delegation, which plays an important role’. In addition, distinguished guests play the role of ‘session chairpersons’. They are Ecolint alumni ‘who are used to presiding in conferences’. Thanks to their leadership roles within the UN system and in NGOs, these guests represent both role models and ‘authority figures’ for students: Serguei Ordzhonikidze, no less than the UN Secretary-General in Geneva; Dennis McNamara, Director of the United Nations Inter-Agency Internal Displacement Division, Rajagopalan Sampatkumar, Professor and Secretary-General of the International Society for Human Values (see Supplemental Annex, program). The aim is to have ‘figures that the students cannot contest’, explained Jean-Marc, ‘because when you have someone who is 45 years old and you say, here is the director of a department at the International Labour Organisation, or something like that, a teenager who is 15, 16 or 18 years old won’t say no, I disagree’.

The ritual’s organizers designed the rules with the following guests in mind: ‘Insofar as we bring people who have ten, fifteen, twenty years work experience with the United Nations – they know what it is like to run a conference with 200 delegates, we don’t have to tell them how to do things’. ‘Speaking out to defend a country’s position in front of people of this ‘caliber’ is not the same as under the familiar eyes of teachers or less experienced university students – as it was in the SUN’, he insists. This further emphasizes the hierarchical relationship between ‘delegates’ and ‘authority figures’ in the ritual: adults occupy a high position in the assembly hall. They are physically apart, lined up on the stage in front of everyone. The teachers who make up the ‘Steering Committee’ sit alternately in the three positions at the head of the ‘Assembly’. Together, they play the roles of ‘presiding officer’, ‘secretary general’ and ‘secretary of the Assembly’. Their quasi-juridical administrative practices consist in granting the right to speak to ‘delegations’ in a formal language. For example, ‘Ladies and gentlemen, honorable delegates, we will do what we can to accommodate you’. Guest speakers join them during the keynote, opening and closing speeches.

Finally, the organizers set up a control system that is not found in the real ‘Assembly’. This involves ‘filtering’ messages to prevent ‘delegates’ from sending each other personal notes. By circulating written messages between the ‘delegations’, the messengers contribute to a silent and continuous dialogue between the teams. They also forward messages to the executive committee. Thus, an impressive silence reigns in the Assembly hall thanks to this device. When the headset is removed, one only hears the noise of those who move from one delegation to another transmitting messages. All these pedagogical measures contribute to creating a favourable ‘atmosphere’ and put students in a professional situation as delegates as realistically as possible.

### Elected to debate: The process of selection

By what means are these high school students taught to act like delegates? While the regulations leave the ‘session presidents’ a wide margin of manoeuver, this is not the case for students. The teachers who organize the ritual strongly regulate the students’ discourse. The form of the ‘debate’ and the way in which the ‘delegates’ intervene are at the heart of the rules. Surprisingly, the debate opens and ends with a ‘minute of silence’ devoted to prayer or meditation. The organizers explain that the minute of silence is in honour of Robert J. Leach who was a ‘Quaker’ (see Supplemental Annex, SLN rules, p. 12). Furthermore, to request the ‘right to the floor’, the ‘delegates’ raise the sign above their heads to the attention of the governing committee, a gesture reminiscent of the hand raised in class. The committee then grants this ‘right’ in a ceremonial way: ‘honorable delegation from China’, for example, ‘you have the right to the floor’. In return, a solemn thank you accompanies all speeches. These forms and formalities sanctify ‘debate’ as an institutional rite, in Bourdieu’s sense.

Indeed, the form in which ‘delegates’ take the floor is strictly defined and limited in time: ‘presentation of a resolution and opening of the debate’ (7 min), ‘right to the floor’ (2 min), ‘point of information’, ‘right of reply’ (2 min), ‘point of order’, ‘amendment’ and ‘final speech’ (5 min). A requirement of great brevity defines these speech forms. Students are often reminded to be ‘extremely concise’. Exceeding the maximum time allowed to them means losing the ‘right to the floor’ (see Supplemental Annex, SLN rules, p. 13).

Regulated and formalized speech is therefore at the heart of the rite. Few students have the ‘talent’ of the American delegate that Jean-Marc considers to correspond to his expectations. The teachers select students who participate in the event in three stages. First, before they take part in the ‘Assembly’, the Committee elects 262 ‘delegates’ based on the ‘resolution’ they have drafted. In return, they get a seat in the assembly hall and a country to represent. Enrolment in Ecolint is therefore not enough. Students whose parents are accustomed to this type of work start one-step ahead of others. Not all the ‘resolutions’ make their way up to the podium: eight authors present only the best four. The resolution presented first is the one that the teachers judge the best out the four. This procedure determines the ‘quality of the debate’ that follows each of the four presentations, explains Jean-Marc. The elected delegates help others, by example, to rise to their level.

When a ‘delegation’ presents its ‘resolution’, the ‘delegates’ who compose it change places in the hall; they go up to the stage, to the left of the ‘Secretary General’. The rite separates these students from the others, bringing them closer to the adults; it raises them higher in the space of the hall. After separating the 262 ‘delegates’ from the other students who express interest in participating, the rite separates the eight best from those who earned a seat in the Assembly. The rite thus solemnly proclaims these students’ difference in front of all. The election of the best is all the more effective when it elevates them above all others. Better than naming by means of signs external to the body such as the badge, the seat and the sign, this ultimate act of consecration is also the most lasting.

While the effect of the rite is durable, the sense of legitimacy it reinforces among high school students also has immediate effects. Thus, the elected ‘delegations’ could obtain a ‘point of information’ to pose the Secretary General of the International Society for Human Values such a trivial question as: ‘What is your favorite Indian dish?’ While teachers remind them that this is not the place for such comments, other ‘delegates’ also used their ‘right to the floor’ to make others laugh by slipping: ‘We would like to thank the student council for the crazy party we had last night’.

Being elected to participate in this miniature ‘Assembly’ modifies the self-representation of the 262 elected students. Moreover, the elected students’ absence from the classroom for 2 days is significant for their classmates. As for the elevation of the top eight, it allows them to cultivate a high idea of themselves not only in front of their peers, but also before their teachers and the eminent guests of the Committee. All this reinforces their difference. It is therefore by a double act of separation, both physical and symbolic, that the rite consecrates the best and most legitimate delegates as ‘being international’.

### Excellence is talent

On the first day, during the morning session, the ‘delegation of Israel’ did not register on the list of speakers, reports Jean-Marc. As a result, the ‘Israeli delegates’ were cut off every time they tried to speak. They were well aware of the rules: when a ‘delegate’ considers that his country or organization is concerned by what was said during an intervention, he may request a ‘right of reply’ to contest the remarks of the previous speaker (see Supplemental Annex, SLN rules, p. 13). They had requested ‘rights of reply’ without success. Unable to express themselves despite their efforts, they voiced their frustration. Then, one of the teachers suggested they ask for an ‘information point’ during the final speech. They took his advice and then took advantage of it so that it became a real ‘right to the floor’. Israel had done very well, explains Jean-Marc because the most difficult exercise is the ‘right of reply’. They were exemplary of the ease that teachers expect from ‘delegates’: ‘[…] when you have students doing this spontaneously, that’s fabulous, because that means they have integrated the mechanism. That is possible here with us; it is not possible in other contexts’.

With this example in mind, we may ask how students ‘integrate the mechanism’: what is required, considered convincing and excellent by the organizers? The conditions that teachers create are not everything: mastery of content and the required forms are necessary but insufficient for students to shine. According to Jean-Marc, it is the quality of the students that makes this role-play unique and exceptional as compared to others. The simulation requires an emotional investment from the students both in writing the ‘resolutions’ and in defending them during the ‘debate’. Indeed, Jean-Marc insists on the whole emotional side of learning, at the expense of a thorough knowledge of the issues at stake:

‘[…] What interested us was the feeling you have when you are in the assembly, so you belong to this great exchange, which has great debates that require you to be *involved*. […] To be *in* a context, where we are not negotiating a comma, where we are not negotiating with 25 thousand references to very specific texts […] we are rather trying to recreate the moment when you represent a country. You must have empathy for what this country is doing, thinking, saying, and you have to be able to translate that, you have to be able… to transpose it’.

**Figure 2.**
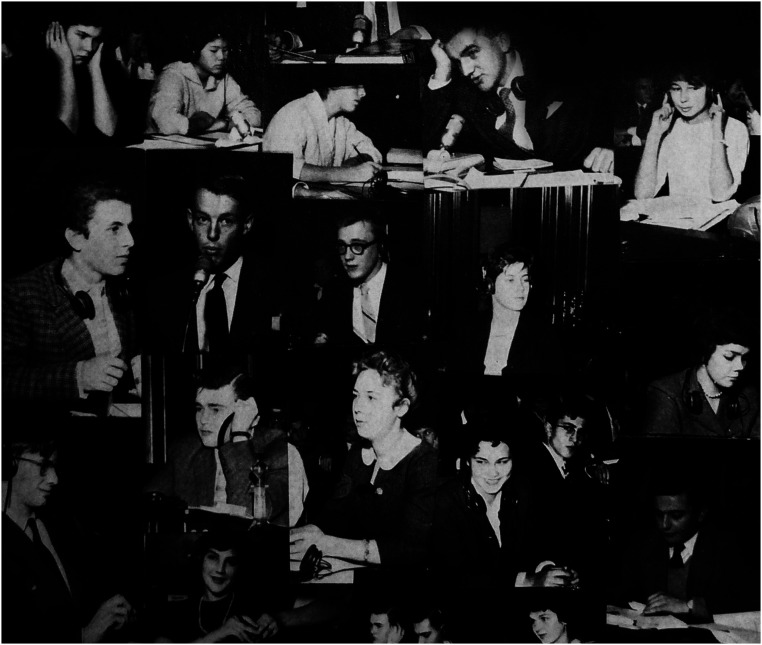
Collage of students photographed at the SUN in 1959 and published in the Ecolint Yearbook the same year. Source: Archive Jean-Jacques Rousseau (AIJJR), Fonds Ferrière, E.IV.9, 1.2.1. University of Geneva, Faculty of Psychology & Education Science.

More than rebuilding its identity, the SLN stands out from other simulations of the UN in that its organizers seek to draw an invisible boundary between ‘us’ and ‘them’. Jean-Marc portrays the SLN as the most legitimate, always on the right side of this boundary. In this context, teachers consider students convincing when they are able to overcome their political opinions. Thus, Jean-Marc tells me that he finds very convincing as an American delegate a student whom he perceives in reality as ‘extreme left’ and ‘totally anti-American’. ‘And that’s ideal’, he explains, ‘that’s someone who is here and who, because there is the context of the SLN, puts himself in the opposite camp, and does it with talent’. Even if the UN context requires many adjustments to specific rules, students must be comfortable when addressing the topics of interest (Figure 2). The SLN recognizes excellence as ‘talent’, which is indeed a way of defining it as indefinable. The institutional rite thus validates a process of socialization begun by other institutions like the family.

While some testify having ‘integrated the mechanism’ through the spontaneity of their discourse, others have more difficulty. Teachers often have to remind them to say ‘Mr. President, honorable delegates’ each time they speak. The pedagogical tone with which they add these reminders at the end of their sentences sharply contrasts with the formalism of their language. These reminders sometimes take the form of a disciplinary warning. For example, all the ‘delegates’ had burst out laughing when a ‘member of the governing committee’ pounds from the platform that it is forbidden to remove the badge, leave the room during the sessions and be rude: ‘you cannot be rude to other delegates or I shall come talk to you’.

As in the classroom when students make fun of the one who plays smart but does not know how to answer a question during the lesson, during the role-play they make fun of those who play the ‘delegates’ but do not rise to the task. According to Jean-Marc, it is because ‘they think it could not happen to them’. If the administrators take particular care to explain the rules of the ‘debate’, it is because these practices are least left to the ‘orchestration of the *habitus*’ (Bourdieu, 2000: 314), that is, to the spontaneity of regulated practice, according to precepts inculcated in advance. The *habitus* refers to the rules and codes of conduct of a social environment that an individual has learnt, incorporated and forgotten as such (Bourdieu, 1980a: 56). Having forgotten that what is incorporated has been imposed on them from the outside, individuals consider their behaviour as constituting their personality. Clarifying the rules of the SLN therefore brings in line those who have not yet fully integrated them, with the aim that following them becomes a second nature.

The closing speech is pronounced by Ecolint’s Director General, Dr. Nicholas Tate.^30^ It is addressed to the public not only as ‘honorable delegates’ and as students, but also as adults, as ‘citizens of their nations and citizens of the world’ and as ‘future senior national or international officials and employees of large multinational corporations’. However, before becoming world leaders, he ends by encouraging them to project themselves as future university students: ‘And when you apply to universities, tell them that this is one of the rather special things you have been involved in during your stay at the School’. Closing the simulation ceremony, the Director suggests the rite these students have undergone will distinguish them upon entry into institutions of higher education and beyond.

Indeed, almost all the students enrolled in Ecolint belong to the social categories that constitute the reference universe of this discourse, suggesting that the ‘world’ outside the School is limited to the mentioned career possibilities. Indeed, these correspond to the students’ fathers’ occupations (cf. Social composition of School). It is as if certain professional categories – that is, other levels of the national public sector or the professions – were excluded from the scope of possibilities to which students could aspire. This normative discourse therefore tells students: ‘become who you are’. It contains the temptation of those who are inside, on the right side of the mental and social boundary set by the rite, to get out, to separate and to downgrade.

### A privilege that reinforces the primacy of the nation-state

While no one has explicitly defined what it means to be ‘international’, the ethnographic study of the SLN has made it possible to examine the socially distinctive practice of representing and embodying a nation-state that the institutional rite dubs ‘international’. These high school students had spent two intense days mingling with international civil servants, who were sometimes also former students of their school. ‘Authority figures’ have addressed them as a future ‘elite’ who will have the capacity to think and act on a global scale. While they embodied delegates for two consecutive days, they were designated as ‘citizens of the world’, and ‘guardians of human civilization’. It is within the walls of the *Palais des Nations* that they were led to reveal themselves to a select audience. They dressed, stood and spoke as ‘delegates’ to the ‘General Assembly’. Some felt more comfortable than others. Yet to ‘become international’ as a group, which they already were in view of the social composition of the school, Dr. Tate reminded the students that they must not lose their individual national identities. Professor Sampatkumar, too, closed his speech on this moralizing advice much applauded by the ‘Assembly’, stressing that ‘You should not start being global citizens unless you are very sure of your national identity’.

Contrasting the interviewees’ rationalization of the educational objectives behind the SLN rules with and their accounts of the selection process and my observations, I find that the teachers who made the rules of the game attribute a socially distinctive meaning to a widespread educational practice of role-play games. It enacts the established order of nation-states all the more effectively that the ritual display of ‘everyday nationalism’ is endowed with an educational purpose.

The rite reinforces students’ national identity by instituting ‘being international’ or ‘global citizenship’ as a privilege embodied in a specific set of practices, bodily techniques and dispositions. It does this in two distinct ways, summarized as follows: (1) it transgresses the established social order based on ethnic self-identification and categorization by others and (2) it reinforces the students’ self-understanding along the lines of ethnicity based on nationhood. In the broader context of ‘international schooling’, the rite grants itself the extraordinary and exclusive privilege of transgressing national identity. Highly selective in terms of participating schools and students, it draws a symbolic, class-based boundary between those who are worthy of this transgression and those who are not. An analogous boundary separates the international from the national and local and, more broadly, the social imaginary around the figure of the ‘expat’ from that of the ‘immigrant’.

The everyday nationalism displayed by the rite is identified as international based on privilege. It contains a strong symbolic dimension, which appears all the more significant when we consider what Marcel Mauss called ‘bodily techniques’ (Mauss, 1950). Indeed, during the SLN, ‘being international’ means being free to transgress national identity based on belonging to a privileged social milieu. By making students embody delegates, the rite displays their social privilege, which teachers interpret as ‘skill’, ‘capacity’, and ‘talent’. In their discourses, the selected few appear to naturally have what it takes to ‘become who they are’. It is by instituting the distinguishing characteristics of the selected group of students as criteria for inclusion and ‘excellence’ in the SLN that the rite legitimizes and makes them appear as second nature, thereby naturalizing social advantages. As Khan has pointed out, the key to learning privilege is embodied ease and how well it is learnt is determined in situations of interaction (Khan, 2010). In the case under study, these interactions are intensified in the 2-day role-play. Teachers encourage and constrain students to exercise the capacity to distance themselves emotionally from national self-identification, and this is then attributed to being ‘international’ and instituted as the difference that distinguishes them as ‘global citizens’ from the unworthy ‘others’ who are viewed as incapable of taking such distance.

Taking the privilege to transgress national identity, however, does not mean dismissing it. On the contrary, by codifying the transgression, the rite reinforces the primacy of the nation-state as a principle of identification and categorization (Brubaker and Cooper, 2000). Moreover, it reaffirms the legitimacy of political internationalism, which leads the students to perceive themselves as representatives of their nations. At the SLN, socio-economically advantaged youth learn that much as they are worthy of the privilege to transgress it, national identity is an integral part of them, that is, that it constitutes their essence, so to speak. Reaffirming symbolic boundaries, the SLN ritually safeguards this essentialist conception of nation-states and thus inculcates lasting dispositions that consecrate the ordinary uses of ethnic self-identification and categorization by others in the established social order.

## Supplemental Material

sj-pdf-1-eth-10.1177_14661381221082909 – Supplemental Material for Becoming ‘international’: Transgressing national identity and everyday nationalism as a ritual for class identification Supplemental Material, sj-pdf-1-eth-10.1177_14661381221082909 for Becoming ‘international’: Transgressing national identity and everyday nationalism as a ritual for class identification by Leonora Dugonjic-Rodwin in Ethnography

## Notes

1. They include national and regional political organizations, such as the United Arab League and the European parliament, the ‘Model EU’.

2. On global fields, see Buchholz (2016); Sapiro (2018).

3. This article is only partially reproduced in Bourdieu’s books that have been translated into English such as *The State* Nobility and *Language and Symbolic Power*, hence my translation.

4. Using Andreas Wimmer as an analytical guide, I consider ethnicity to subsume race and nationhood, which is predominant in such a school setting (Wimmer, 2008).

5. On political internationalism, see Mazower (2009); Sluga and Clavin (2017) and its application to pedagogy, see Dugonjic-Rodwin (2014, 2022); Hofstetter and Schneuwly (2013).

6. I conducted quantitative analyses on the social composition of the School by cross-referencing public statistics dating back to 1969 with a sample established for two grade years (1976 and 2000) based on the enrolment forms found in the School archive.

7. For a detailed argument in favour of this use of interviews, see Bourdieu (1981).

8. Source: https://www.ecolint.ch/campus/la-grande-boissiere, consulted on 28 November 2019.

9. SRED is part of the Department of Public Education of the State of Geneva and has a research mission on the education system to produce statistical information ‘useful for its management and knowledge’. *SRED, Research and Activities* 2005–2006, p. 3.

10. Based on the exchange rate of 19 November 2019, see https://www.ecolint.ch/admissions/tuition-and-fees.

11. I refer to father’s occupation because the quasi-totality of mothers at the School are limited to domestic work as ‘homemakers’ or ‘trailing spouses’ (Dugonjic-Rodwin, 2022).

12. Since 1946, the Education Grant has ensured that any organization within the United Nations system pays 75% of the school fees to the children of expatriate civil servants at the ‘professional’ level.

13. On global language hierarchies, see De Swaan (2001).

14. Interview with Roger, 17 October 2007: French teacher at Ecolint since 1993, Bachelor of Arts degree from the University of Geneva, Switzerland, age 39.

15. Interview with Vladimir, 11 October 2007: Math and TOK teacher at Ecolint since 1997, Doctor of Mathematics at Lomonossov University in Moscow, Russian, age 56.

16. Interview with Marwane, 2 October 2007: English teacher at Ecolint since 1974, Master of English Paris 10, French, age 61.

17. The following clauses were enshrined in the school’s 2008 website: ‘To help its students to be faithful to their convictions’ and ‘to understand that nationality, gender, religion and culture determine the individual’s starting point’ (Ecolint, 2008).

18. Brochure distributed to participants 2008 and reproduced in Supplemental Annex.

19. Interview with Claude, 20 May 2021: English teacher at Ecolint since 1989 and SLN organizer for 32 years, Bachelor in English Literature, University of York, England, age 65.

20. Interview Claude.

21. Interview with Jean-Marc: History teacher at Ecolint since 1974 and SLN organizer for 13 years, Bachelor of History, University of Geneva, Switzerland, age 45. Unless otherwise indicated, the following excerpts quoted are from Jean-Marc.

22. Interview Claude. Also see Supplemental Annex, Interview extracts 2.

23. Compulsory secondary school approved by the French *lycée*, preparing for the Swiss *Maturité fédérale*, equivalent exam of the French *baccalauréat*.

24. Interview Claude.

25. On continuity between the League of Nations and the United Nations, cf. Clavin (2013); Mazower, (2009).

26. The expression is from Adolphe Ferrière (1879–1960), Leplaysian sociologist and professor at the Jean-Jacques Rousseau Institute, founder of the International Bureau of New Schools in 1899. He was among the founders of the ‘International School’ in Geneva in 1924. Archives of the Rousseau Institute, Fonds Ferrière, E.IV.9 bis, 2.3. Articles, Press clippings. Adolphe Ferrière, Une Société des Nations en miniature: l'École internationale de Genève [corrected proof] 1925, p. 5.

27. Appelby College, Oakville, Ontario, Canada; Arnhem International School, Arnhem, Holland; City Technology College, Birmingham, England; Felsted School, Essex, England; Framlingham College, Suffolk, England; Inter-Community School, Zurich, Switzerland; Lycée International, Saint-Germain-en-Laye, France; New English School, Kowait; Newport Free Grammar School, Saffron Walden, England; The Sultan”s School, Oman; United World College of the Atlantic, Wales; United World College Mostar, Bosnia and Herzegovina; United World College Red Cross Nordic, Norway.

28. Supplemental Annex: SLN brochure.

29. Interview Claude.

30. Prior to leading Ecolint from 2003, Nicholas Tate was advisor to Margaret Thatcher, Principal at Winchester College, a prestigious 14th-century English private high school, Executive Head at the School Curriculum and Assessment Authority (1994–2000) and a member of the National Curriculum Council since 1989. A graduate with a doctorate in history from Oxford, Bristol and Liverpool Universities, he has written seven history textbooks and spent 11 years in public school curriculum administration bodies in England.
