# Supplementary material for: Becoming ‘international’: Transgressing national identity as a ritual for class identification
Source: Ethnography. 2022 Aug 26;25(2):119–41. doi: 10.1177/14661381221082909 (PMC11153022; doi:10.1177/14661381221082909)

Annex

Documents from the participants' booklet 2006: Program and Rules from SLN Brochure

STUDENTS' LEAGUE OF NATIONS

FOUNDED AS "STUDENTS' UNITED NATIONS" IN 1953

Programme of the Thirteenth General Assembly

Geneva, 18th and 19th December 2006, at the United Nations

Monday 18th December

8:30: Doors open (Salle XVII, Pregny Gate, New Building, Palais des Nations)

9:40: Inauguration of the General Assembly: Speech by Mr. Sergei Ordzhonikidze, Director-General of the United Nations Office at Geneva

10:15: First resolution (United States of America)

12:15: Lunch

13:45: Second resolution (Gambia)

15:15: Keynote speech by Mr. Dennis McNamara, Director of the United Nations Inter-Agency Internal Displacement Division (IAIDD)

16:00: Second resolution (continuation)

16:30: End

Tuesday 19th December

8:30: Doors open (Salle XVII, Pregny Gate, New Building, Palais des Nations)

9:30: Third resolution (Democratic People's Republic of Korea)

10:15: Keynote speech by Professor Rajagopalan Sampatkumar, Secretary General of the International Society for Human Values

11:00: Break

11:15: Third resolution (continuation)

12:30: Lunch

14:00: Fourth resolution (United Kingdom of Great Britain and Northern Ireland)

16:00: Closing ceremony, Speech by Dr. Nicholas Tate, Director General of the International School of Geneva

16:30: End

RULES OF THE GENERAL ASSEMBLY

A - PREPARATION FOR THE GENERAL ASSEMBLY

Objective
The objective of the General Assembly is to conduct debates on resolutions prepared and chosen in accordance with the present Rules.

Official Languages
The official languages of the General Assembly are English and French.

Agenda
The Agenda of the General Assembly is fixed by the Steering Committee.

Composition of Delegations
1. Member states and official observers are represented by a delegation composed of a maximum of two delegates.
2. A delegate may not represent his / her own country of origin.

B - DRAFTING AND SELECTION OF RESOLUTIONS

General Knowledge
1. Each delegation must have adequate general knowledge of the country or international organisation which it is representing, as well as of the subjects which will be debated in the General Assembly.
2. Each delegation must also know how to draft a resolution.

Required Format
1. The resolutions are drafted in French or English and are translated into the other language; the original version is indicated and is binding.
2. The text of each resolution must be contained on one A4 page.
3. The following information must be shown at the head of the resolutions:
   a) the name of the delegation or delegations presenting the resolution;
   b) the theme;
   c) the subject (title) of the resolution.

Selection
The Steering Committee selects the resolutions to be debated from among those submitted to it by the participating delegations in advance of the General Assembly.

C - THE FUNCTIONING OF THE GENERAL ASSEMBLY

Members
The Administration of the General Assembly is always composed of a Session Chairman and the Secretary General (who is a member of the Steering Committee).

Role of the Chairman and the Secretary General

1. The Chairman's authority is paramount in the General Assembly and cannot be challenged. He / she chairs the debates fairly and impartially, without influencing their content, and observes that the present Rules are applied.
2. The Secretary General assists the Chairman in his / her task.

Calls to order by the Chairman
The Chairman can call a speaker to order if his / her proposals do not deal with the subject under debate, or if he / she is breaking the present Rules.

Abuses and Sanctions

1. If a delegation abuses the regulations and / or ignores the calls to order of the Chairman the latter may, having first warned the delegation, suspend its rights of participation until the end of the debate on the resolution under discussion.
2. If a delegate hinders the smooth running of the debate by his / her behaviour, he / she will be excluded from the General Assembly for the rest of the day.

D - PROCEDURE OF DEBATES

Minute of Silence
At the opening, as well as at the closure of the General Assembly, the Chairman asks the delegates to observe a minute of silence, devoted to prayer and meditation.

= cf. Robert Leach (Quaker)

Terms of address
On obtaining the floor, the delegates must address the Assembly in these terms: "Mister / Madam Chairman, Honourable Delegates..."

a) Debates

Presentation of a Resolution and Opening of Debate

1. At the beginning of each debate the appropriate resolution is presented by one or both delegates.
2. The presentation may last a maximum of seven minutes.
3. After the presentation, only points of information may be requested. All other points are suspended until the opening of the debate.
4. When all points of information have been addressed, the Chairman opens the debate.

Rights to the floor

1. Delegations that wish to speak on one or more specific resolutions must request a right to the floor for each in advance of the General Assembly by contacting the S.L.N. e-mail address. On the basis of the total number of requests per resolution, the Chairman and the Secretary General will compile, with due regard to fairness and balance, an official speakers' list for each resolution. These lists will, at the very latest, be communicated to delegates at the outset of the General Assembly. If there is any time left over after the official speakers' list has been exhausted, the Chairman will consider requests for additional rights to the floor from delegations that have not yet spoken.
2. A right to the floor gives a delegation the possibility of addressing the General Assembly for a maximum of two minutes.

b) Rules of Procedure

Points of Information

1. The purpose of a "point of information" is to request or provide *factual* and *specifically relevant* information as briefly as possible. It is authorised by the Chairman only after the presentation of a resolution, a speech from the floor or a right of reply.
2. The Chairman gives the floor in turn to each delegate who has requested a point of information. The delegates address their factual question or statement to the speaker, who may then reply briefly.
3. Only four points of information are authorised for each speech.

Rights of Reply

1. When a delegate deems that his / her nation or organisation is directly implicated by what has been said during a speech, he / she may request a right of reply.
2. Once a speaker has concluded his / her speech and has replied to any points of information, the Chairman grants a right of reply to those delegations which have both requested and justified it.
3. The right of reply may be used only to challenge or refute the words of the last speaker and may not last longer than two minutes.

Point of Order

Exceptionally, if a delegation observes a violation of the rules of procedure that has escaped the notice of the Chairman and the Secretary General, it may draw attention to the anomaly by raising a "Point of order". Points of order will be dealt with by the Chairman at the first suitable pause in the proceedings.

Amendments

The Chairman may, at his / her discretion, call for one or more pauses in the debates, in order to allow delegations to contact one another privately and to propose amendments to the resolution under discussion, whereby a consensus may more easily be reached.

Concluding Speech

1. Immediately before the closure of the debate, the Chairman gives the floor to the delegation which presented the resolution. If the resolution is being presented by several delegations, the latter can speak one after another, but the allotted time is divided between the speakers.

2. The delegation has five minutes in which to rectify any errors of interpretation which have arisen during the debate, to announce any amendments it has accepted or to reaffirm and summarise its position.
3. No rights of reply are admissible during the concluding speech, but the Chairman may authorize a limited number of points of information when it is over, if time permits and if he / she considers them useful for the purpose of clarifying the resolution in its final form.

c) Voting Procedure

Closure of Debate

1. When the time allotted for a resolution has passed or if there are no more delegates wishing to speak, the Chairman requests a concluding speech, following which he / she may allow a limited number of points of information, before announcing the closure of debate and the beginning of the voting procedure.
2. No further interruptions are permitted once the Assembly is in voting procedure.
3. Each delegation is entitled to one vote. The vote is taken by roll call.

Majority and Votes Expressed

1. For a resolution to be adopted it must receive a simple majority of the votes registered.
2. According to the present Rules, the registered votes are the total of positive and negative votes, without the abstentions.
3. In case of a tied vote, a second vote is taken. If, on the second vote, there is again a tie, the resolution is rejected.

Interview extracts

1. "One of my great successes was to allocate Cuba to a very bright year 12 student who was very articulate but very reactionary (...) conservative US, really ultra conservative, and ferociously anticommunist. I allocated Cuba- I thought it would be a good educational experience. He was very upset about that and I said it's Cuba or nothing and actually its a great privilege because Cuba is very sought after and that's a mark of confidence in you on my part because Cuba is one of the countries that always has a high profile in SLN debates (...) And I told the student you're very lucky to get Cuba. And he said: 'no, it's really antithetical to what I believe in.' And I said: 'that's the whole point, I want you to investigate Cuba and to represent the Cuban authorities convincingly, I'm sure you will do a good job.' And he did. At the end of it, he said: 'you know Mr... I feel differently about Cuba, I still have my own convictions but I understand better the Cuban perspective on their country, it has changed my mind on what I think and feel about Cuba.' *That's the whole point*, you know, that's the ideal educational outcome." [1]

2. "There were actually no debates. It was a circus. I cannot use language strong enough to condemn what I saw for two or three years before we pulled out. An absolute disgrace…It was appalling. And it still goes on like that, I don't think it's changed. The teachers from the Geneva collèges were totally cynical about it: '*Mais c'est ludique, mais ils s'amusent, mais c'est bien, faut laisser faire*.' Well we pulled out and set it back on the rails, and that's what students like, they wanted to be serious and earnest. Our own students were disenchanted." [2]

Ethnographic Journal & Photographs

[Rewritten ethnographic journal notes, *December 18, 2006*]

I arrived around eight o'clock to discover a long queue at the security post in front of the Palais. The entry procedures put students, teachers and myself in the position of the real participants in a UN conference: security guards, X-ray screening and conference registration form. Once completed, all of us filed in a form in return

---

1 Interview Claude.

2 Interview Claude. Translation of the quote in French: "But it's fun, but they're having fun, but it's good, we should *laissez-faire.*"

for our badges: red for "delegates," black for "messengers" and blue for teachers. At the General Assembly in which we were going to participate, the badge was the first brand that distinguished us from each other. Mine was blue, stating in brackets that I was "alumna." I was more on the side of the teachers than the students, but in reality I did not belong to either group.

I pushed the door of Room XVII open for the second time at the United Nations (UN) headquarters in Geneva. I was one of the students the first time; I am an alumna and a sociologist now. The role-play began a quarter of an hour later. Carefully placed signs on the tables ordered the delegates" seats according to the names of the Member States: Afghanistan, Algeria, Angola, etc. From the podium, I could clearly distinguish the seats, in pairs, bearing the name of a nation-state. High school students in skirts, suits and ties, settled there in pairs. Equipped with microphones and conference-style earphones, they represented a country of their choice. Unlike me when I was in their place, they seemed to take the role-play game seriously.

As a stateless student at Ecolint between 1993 and 1999, I resented its insistence on having us "represent" our nationality. I had ready-made answers that were bluntly critical of what I perceived as the School's "blind acceptance" of the existing hierarchies among languages, nations and nationalities. As a sociologist, however, I returned with a host of questions: Why would a school make its students represent nations? What does it mean to "be international"? What does internationalism signify in this context and how is it practiced?

I was surprised to see high school students playing the "delegates" with what I perceived as "seriousness" and "enthusiasm" because I did not remember being caught in the game when I was in their place. At the time, participating in the simulation meant fun time with my friends outside the classroom; and the messages that were circulating through younger students fulfilled the same function for us as the notes we wrote during class (flirting, bullying, expressing boredom, etc.).

Photos of room XVII at the Palais des Nations

1. Second glass entrance door

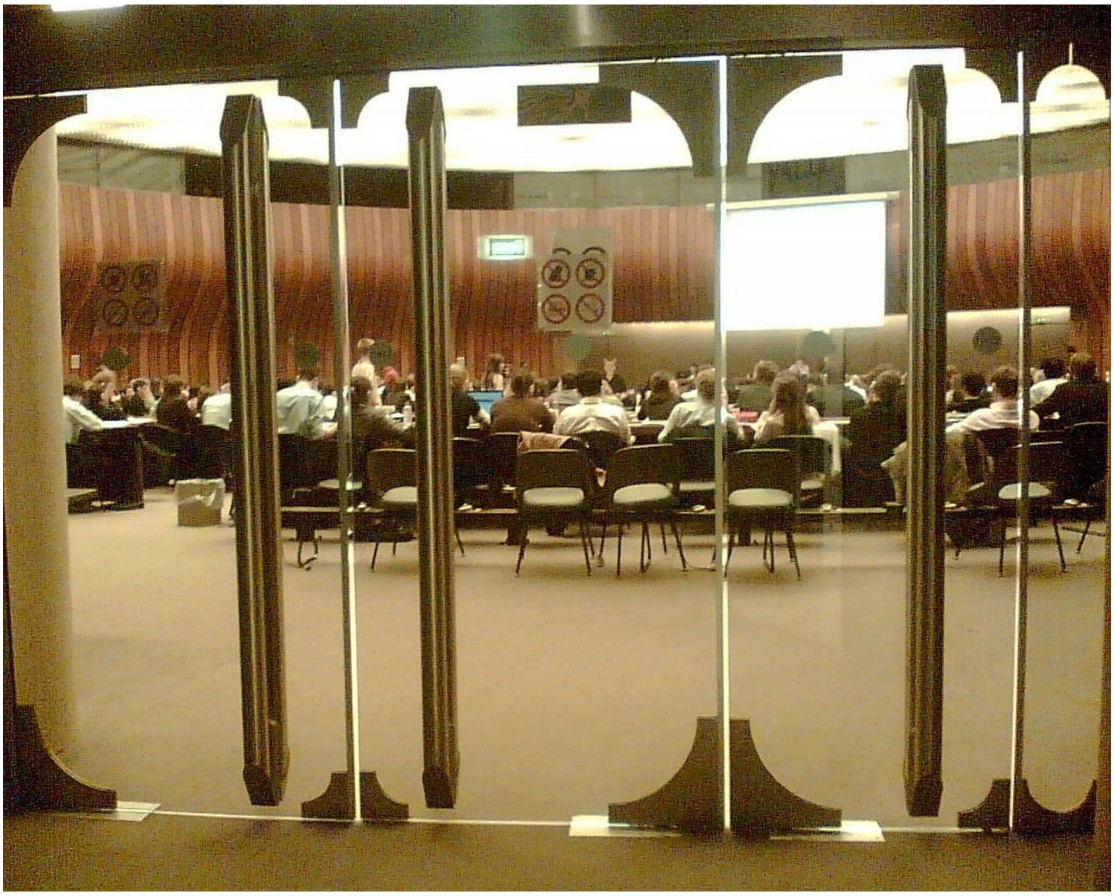

2. Overlooking view during a break.

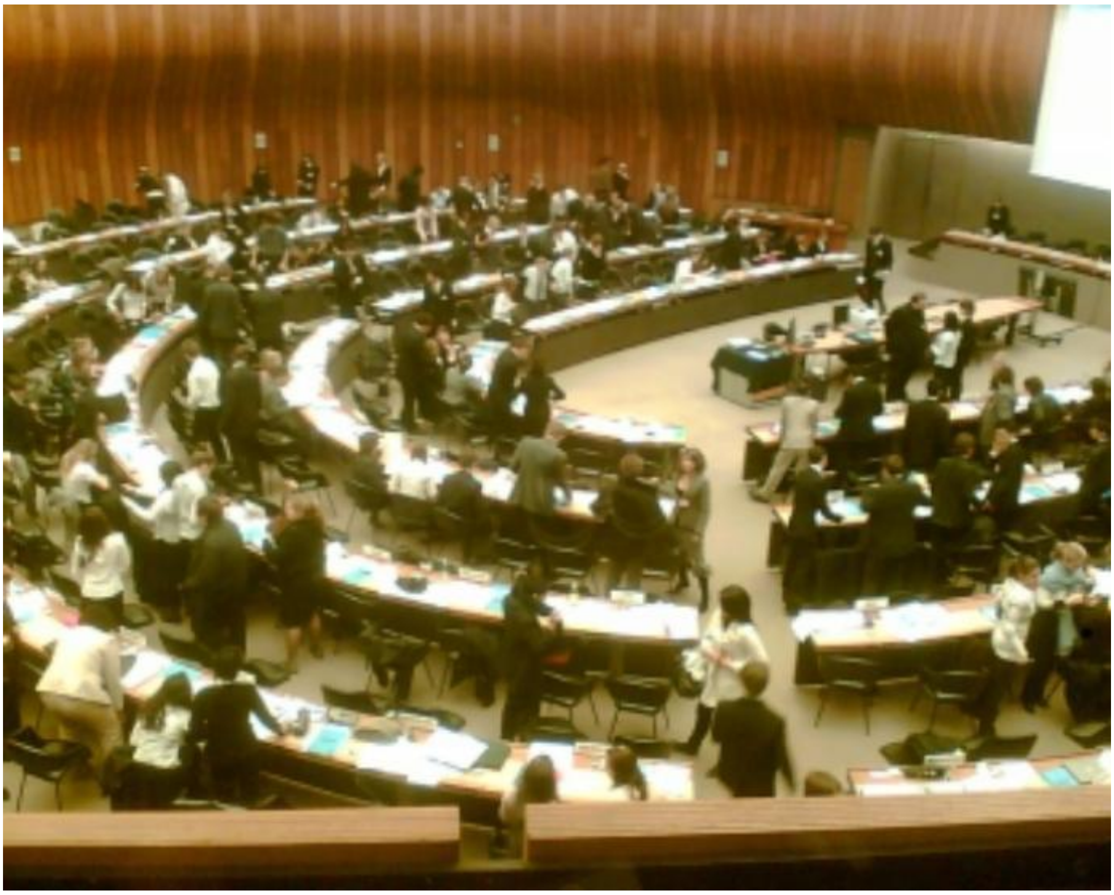

3. View on the empty room and the podium from the entrance

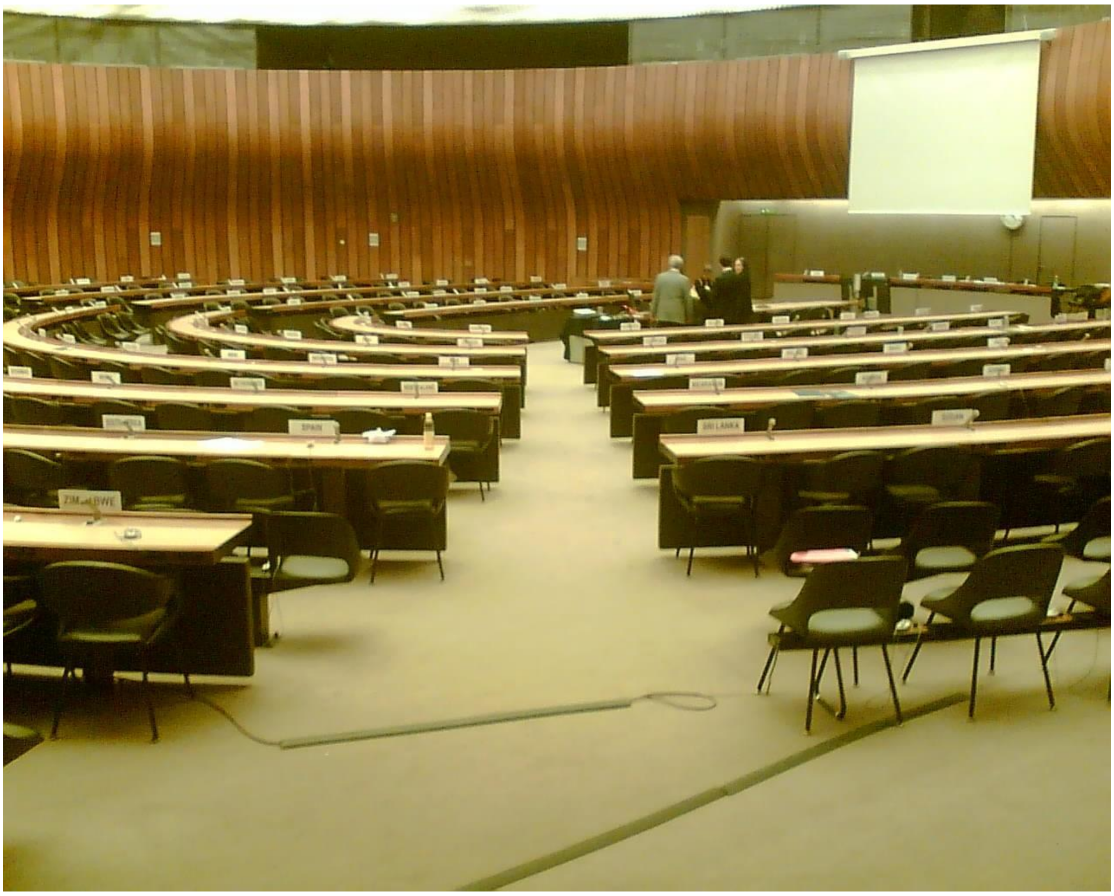

4. A “delegate” raises the sign to ask for the right to the floor.

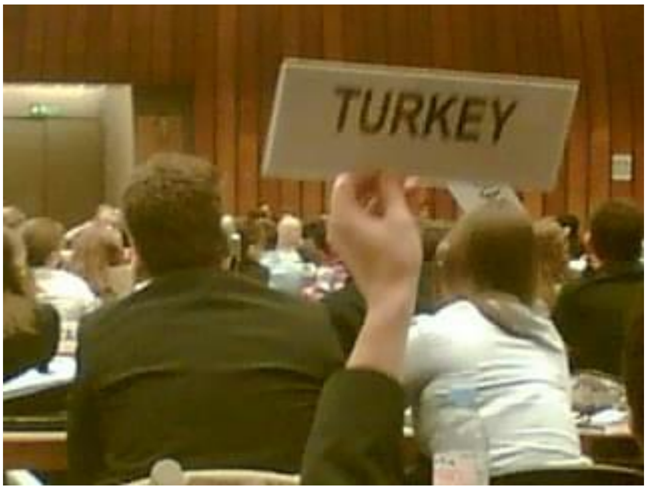

Supplement: sj-pdf-1-eth-10.1177_14661381221082909 – Supplemental Material for Becoming ‘international’: Transgressing national identity and everyday nationalism as a ritual for class identification [file sj-pdf-1-eth-10.1177_14661381221082909.pdf]
